# Fragmentation and Ionization Efficiency of Positional and Functional Isomers of Paeoniflorin Derivatives in Matrix-Assisted Laser Desorption/Ionization Time-of-Flight Mass Spectrometry

**DOI:** 10.5702/massspectrometry.A0101

**Published:** 2022-02-28

**Authors:** Tohru Yamagaki, Kohtaro Sugahara, Kohki Fujikawa, Kazuto Washida

**Affiliations:** 1 Suntory Institute for Bioorganic Research, Suntory Foundation of Life Sciences, 8–1–1 Seikadai, Seika-Cho, Soraku, Kyoto 619–0284, Japan; 2 Nara Prefectural Agricultural Experiment Station, 88 Shijo, Kashihara, Nara 634–0813, Japan

**Keywords:** paeoniflorin, albiflorin, ionization efficiency, fragmentation, MS/MS

## Abstract

Paeoniflorin and albiflorin, which are functional isomers, are the major constituents of an herbal medicine derived from *Paeonia lactiflora*. Those functional isomers and their galloylated derivatives, which are positional isomers, were studied by matrix-assisted laser desorption/ionization–tandem mass spectrometry (MALDI-MS/MS). The resulting mass spectra are discussed based on the fragmentation patterns of the sodium adducts. The product ion spectra of 4-*O*-galloylalbiflorin and 4′-*O*-galloylpaeoniflorin differed, even though they were positional isomers. The fragmentations of the ester parts were influenced by the neighboring hydroxyl groups. The ionization efficiency of the sodium adduct of albiflorin was higher than that for paeoniflorin. These results indicate that the carboxylic ester group has a higher affinity for sodium ions than the acetal group, which can be attributed to the carbonyl oxygen being negatively polarized, allowing it to function as a Lewis base.

## INTRODUCTION

**Figure 1:**
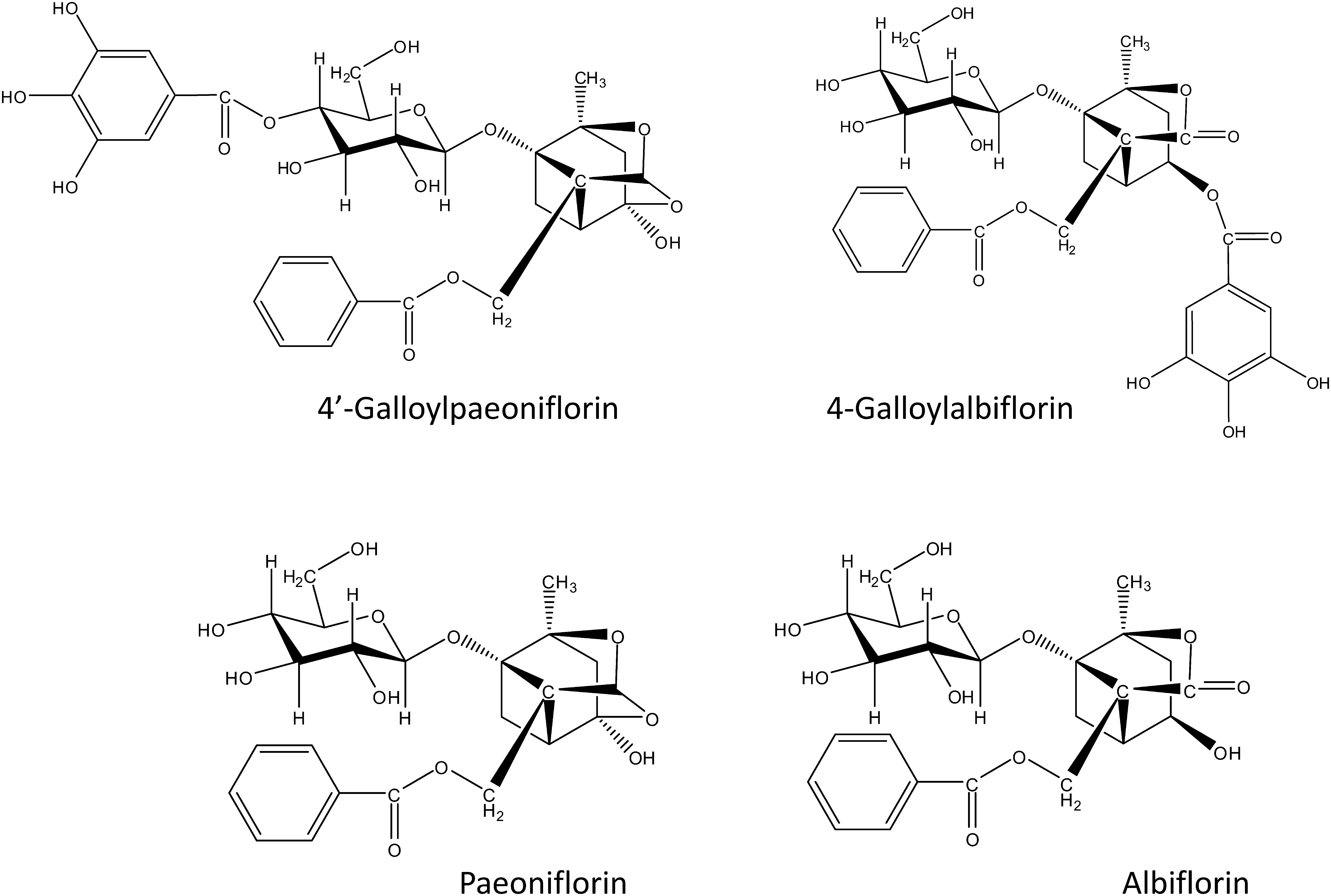
Fig. 1. Structures of 4′-galloylpaeoniflorin, 4-galloylalbiflorin, and paeoniflorin, and albiflorin.

The roots of *Paeonia lactiflora* are used in traditional herbal medicine for the treatment of female hormone-related issues, including menopause, menstruation, blood problems, and general body pain.^1–4)^ Paeoniflorin and albiflorin, terpene functional isomers that contain D-L-β-glucopyranosyl and benzoyl groups, are the major constituents of the roots of *Paeonia lactiflora*. Two new galloylated monoterpene glycosides (4-*O*-galloylalbiflorin and 4′-*O*-galloylpaeoniflorin) (Fig. 1) were recently isolated from the roots of *Paeonia lactiflora* that was obtained in the Nara Prefecture of Japan.^4)^ These compounds were galloylated positional isomers and were reported to exhibit androgen-modulating and receptor-binding activities.^4,5)^ The chemical constituents of *Paeonia lactiflora* roots, including paeoniflorin, albiflorin, and their derivatives, were investigated *via* high-performance liquid chromatography,^6)^ electrospray ionization mass spectrometry (ESI-MS), and tandem mass spectrometry (MS/MS).^7–10)^ Molecular imaging of the metabolites in these plant tissues using an imaging MS technique was recently used to examine their functions.^11–13)^ The imaging MS was used to determine where the metabolites were localized in the plant tissues, since it is difficult prepare antibodies to such small molecules. Matrix-assisted laser desorption/ionization mass spectrometry (MALDI-MS) is one of the best methods for characterizing small molecules. Therefore, the plant metabolites should be studied well in their ionization and fragmentations in MALDI-MS.

We initially attempted to distinguish between the positional isomers of 4′-*O*-galloylpaeoniflorin and 4-*O*-galloylalbiflorin using their MALDI-MS/MS spectra, and then evaluated the ionization efficiency of the functional isomers of paeoniflorin and albiflorin based on their sodium adduct ions.

## EXPERIMENTAL

### Materials

HPLC grade methanol and ethanol, formic acid, and sodium trifluoroacetate (NaTFA) were purchased from Nacalai Tesque Inc., Kyoto, Japan. MALDI grade 2,5-dihydroxybenzoic acid (DHBA) was purchased from Sigma-Adlrich Inc. (St. Louis, MO, USA). Albiflorin was purchased from ChromaDex Inc. (Irvine, CA, USA). Paeoniflorin was purchased from LKT laboratories, Inc. (St. Paul, MN, USA). Rutin, a flavonoid glycoside, was purchased from Extrasynthese S.A. (Genay, France).

### Samples

4-*O*-galloylalbiflorin and 4′-*O*-galloylpaeoniflorin were isolated from the roots of *Paeonia lactiflora* (Paeoniae Radix), which were collected in the Nara Prefecture of Japan. Rutin was used as an internal standard for estimating the ionization efficiency of paeoniflorin and albiflorin in MALDI-MS. A 1 mg/mL stock solution of rutin was prepared in methanol. A 10-mg/mL solution of DHBA in 50% ethanol was prepared as a MALDI matrix.

### Mass spectrometry

The MS and MS/MS product ion spectra (in the positive reflectron mode) were obtained using an Ultraflex III MALDI-TOF/TOF MS instrument from Bruker Daltonics GmbH (Bremen, Germany). The YAG-laser wavelength used was 355 nm. For the MS experiments, the accelerating voltage was 25 kV. For the MS/MS experiments, the initial accelerating voltage was 18 kV, which was later increased to 19 kV. The product ions were generated by post-source decay.

### Estimation of ionization efficiency of paeoniflorin and albiflorin

The MALDI-MS ionization efficiencies of the analytes as a function of their concentration were estimated from their peak intensities, which were normalized against that of rutin or the internal standard. The concentrations of each analyte studied were 5, 10, 20, 40, 80, 100, and 150 μM. The concentrations of rutin and NaTFA (sodium dopant) in the samples were 100 μM and 1 μg/mL, respectively. The matrix solution was comprised of 10-mg/mL DHB and 0.1% formic acid in 70% methanol. Each MALDI-MS spectrum was generated from 500 scans. Peaks corresponding to paeoniflorin and albiflorin were both observed at *m*/*z* 503. They were detected as the sodium adduct ions, [M_pae_+Na]^+^ and [M_alb_+Na]^+^, respectively. Their peak intensities were normalized against the rutin peak [rutin+Na]^+^ at *m*/*z* 633. The peak intensities for paeoniflorin, albiflorin, and rutin were expressed as *I*_pae_, *I*_alb_, and *I*_rutin_, respectively. The ratios of *I*_pae_/*I*_rutin_ and *I*_alb_/*I*_rutin_ were plotted against each analyte concentration. The data presented are the averages of three measurements and the included error bars represent the standard deviations.

## RESULTS AND DISCUSSION

### Fragmentation of galloylated positional isomers of 4-*O*-galloylalbiflorin and 4′-*O*-galloylpaeoniflorin

**Figure 2:**
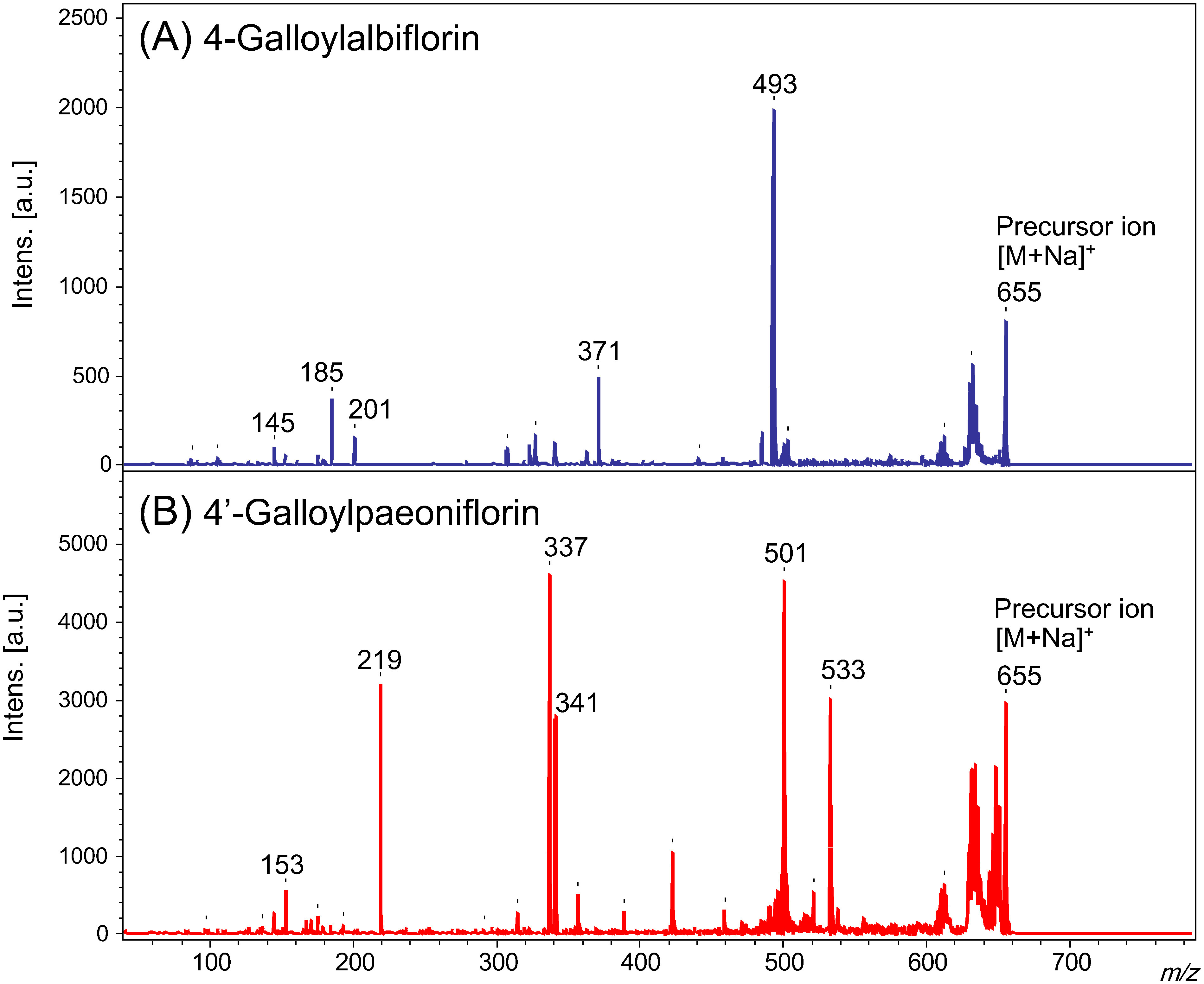
Fig. 2. MALDI LID-MS/MS spectra of 4-galloylalbiflorin (A) and 4′-galloylpaeoniflorin (B).

**Figure 3:**
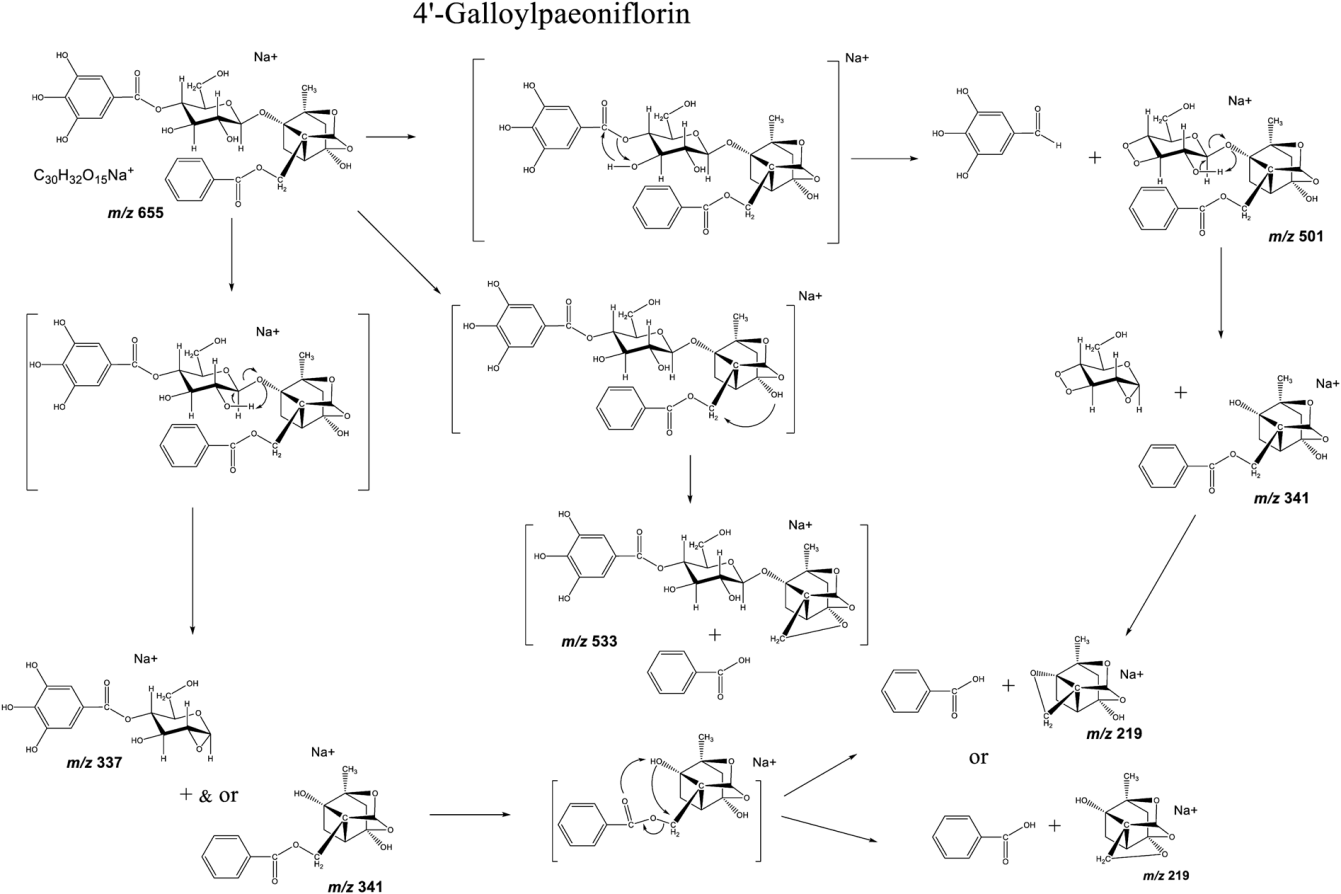
Fig. 3. Proposed fragmentation pathway of 4′-galloylpaeoniflorin.

The MALDI-MS/MS spectra of 4-*O*-galloylalbiflorin (4-GA) and 4′-*O*-galloylpaeoniflorin (4′-GP) show the formation of sodium adducts ([M+Na]^+^) at *m*/*z* 655 (see Fig. 2). In the spectrum of 4′-GP, product ion peaks at *m*/*z* 501 and 533 were observed, indicating that the galloyl (=154 u) and benzoyl (=122 u) groups had been removed from the molecule (Figs. 2B and 3). However, these product ions were not observed for 4-GA (Fig. 2A). During the fragmentation of 4′-GP, the galloyl group located next to a hydroxyl group in the glucose unit was removed (Figs. 2B and 3). Meanwhile, the benzoyl group located next to a hydroxyl group in the paeoniflorin aglycon was removed. The glycosyl bond of the glucose unit was cleaved with the aid of the C-2 glucosyl hydroxyl group (Figs. 1 and 3). This fragmentation has been previously reported in sugar chain fragmentation in low-energy collision-induced dissociation ESI-MS/MS.^14,15)^

**Figure 4:**
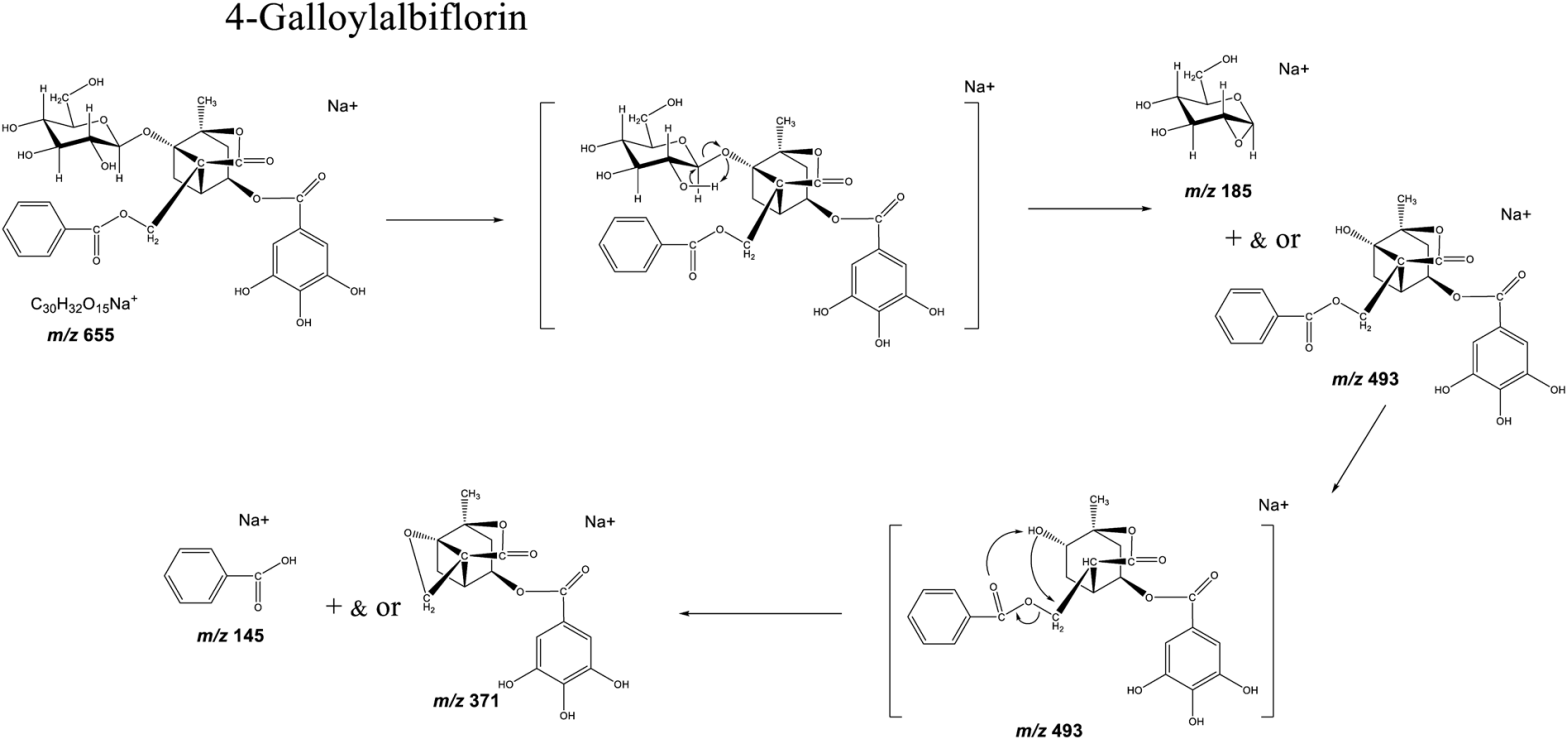
Fig. 4. Proposed fragmentation pathway of 4-galloylalbiflorin.

In 4-GA, no hydroxyl groups are located close to the benzoate or galloyl groups (Figs. 1 and 4). Therefore, the glycosyl bond was cleaved first due to the neighboring hydroxyl group (C-2) in the glucose unit. This resulted in an easier access to the new neighboring hydroxyl group in the glucose unit, further leading to the removal of the benzoyl group (Fig. 4). However, the galloyl group was not removed due to the absence of a neighboring hydroxyl group. In summary, the fragmentation of esters at the galloyl, benzoyl, and glucosyl groups of the analytes were induced by a neighboring hydroxyl group. These findings are critical for understanding the MALDI-MS/MS spectra of paeoniflorin and albiflorin derivatives, which contain the same structural units.

### Ionization efficiency of albiflorin and paeoniflorin in MALDI

**Figure 5:**
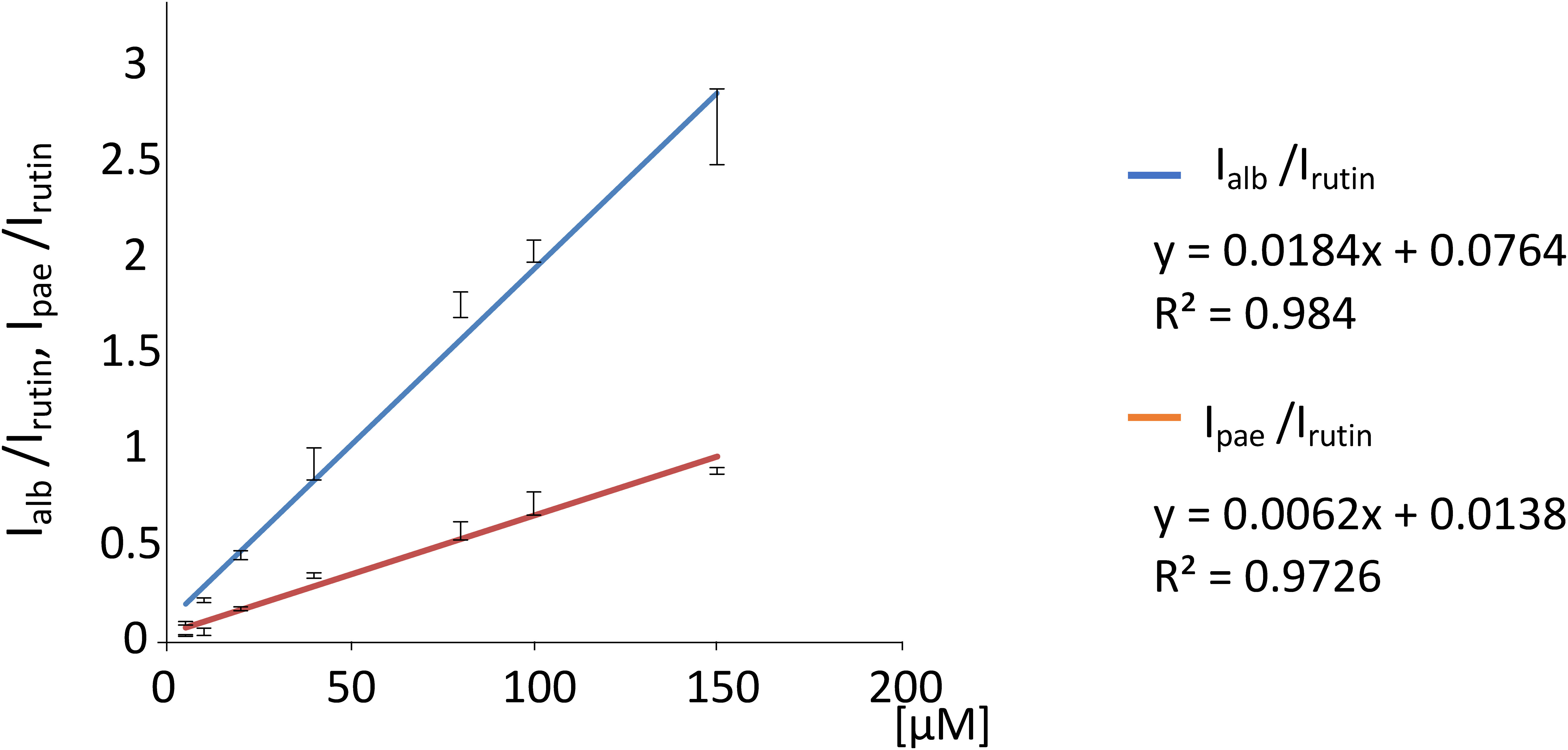
Fig. 5. Plots of peak intensities of paeoniflorin and albiflorin to estimate their ionization efficiency.

The sodium adduct ion [M+Na]^+^ dominated the MALDI-MS spectra of paeoniflorin and albiflorin. Figure 5 shows the ionization efficiencies of albiflorin and paeoniflorin as a function of concentration. The ionization efficiencies were estimated from the intensities of the [M+Na]^+^ ions, which were normalized using the intensity of the internal standard or rutin (Fig. 5). Interestingly, the relative ion intensity of albiflorin was higher than that of paeoniflorin for all concentrations that were studied, indicating that the ionization efficiency of albiflorin was higher than that of paeoniflorin (Fig. 5). This finding suggests that the carboxylic ester of albiflorin has a higher affinity for the sodium cation than the acetal group of paeoniflorin. The sodium ions were able to more easily attach themselves to the oxygen atom due to the stronger polarization in the carboxylic ester than that in the acetal group. A similar situation was observed with the acetamide group.^16)^

*I*_pae_ and *I*_alb_ indicate the peak intensity of paeoniflorin (pae) and albiflorin (alb) at *m*/*z* 503 as a sodium adduct ions. Their peak intensities were normalized to the internal standard rutin peak at *m*/*z* 633 (*I*_rutin_). The ratios of *I*_pae_/*I*_rutin_ (blue line) and *I*_alb_/*I*_rutin_ (red line) were plotted against each concentration for the estimation of their ion efficiency. These data represent the average of three measurements and the error bar indicates the standard deviation.

Li *et al.* reported on the results of density functional theory calculations (DFT) on the negative charges of the oxygen atoms of paeoniflorin and albiflorin^10)^ Their results indicated that the oxygen atoms of the sugar hydroxyl groups and benzoyl esters had lower negative charges than those of the aglycon moieties. This indicates that the sodium cation has a high affinity for the sugar moiety and/or benzoyl esters, as reflected by DFT. However, the difference in the ionization efficiency between albiflorin and paeoniflorin cannot be explained by the location of the sodium cation in the molecule. The observed difference can be attributed to differences between the structures of albiflorin and paeoniflorin. Therefore, the affinity of the carboxylic ester in albiflorin appears to be stronger than that of the acetal group in paeoniflorin for the sodium ion. It was recently reported that the acetamide groups had a much higher affinity for sodium ions compared with the hydroxyl and amino groups in sugars. This caused shifts in the charge center and differences in the product ion spectra and fragmentation patterns of the sugar chains.^16)^ The acetamide group can be considered to be a key structure for the attachment of an alkali metal ion in the ion source. The carbonyl oxygen atom in the acetamide group was most likely polarized negatively, and showed Lewis basicity. This carbonyl oxygen had a strong affinity for sodium ions.

**Figure 6:**
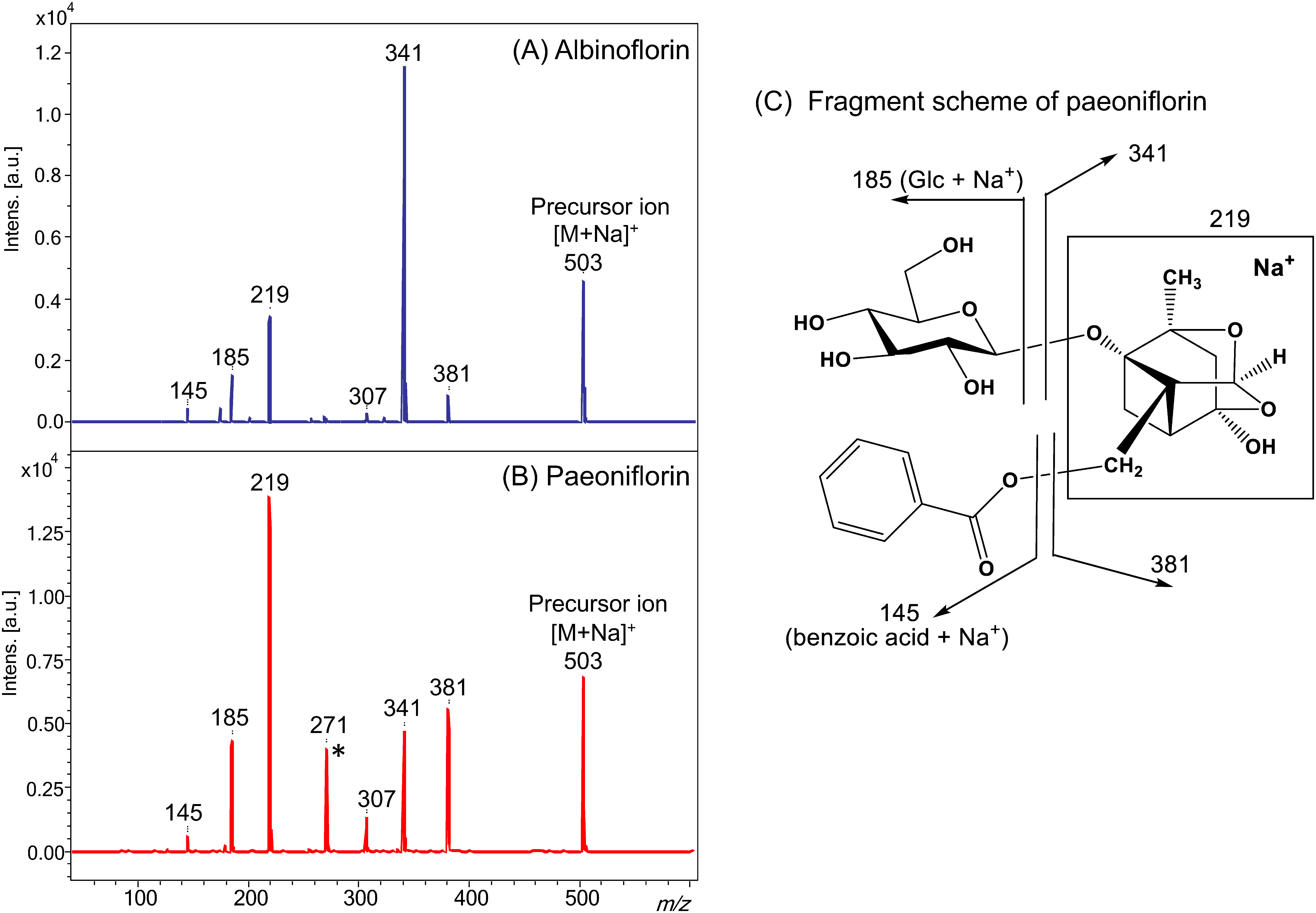
Fig. 6. MALDI LID-MS/MS spectra of albiflorin (A) and paeoniflorin (B). The fragmentation scheme of paeoniflorin (C).

The MS/MS product ion spectra of paeoniflorin and albiflorin are presented in Fig. 6. The concentration of paeoniflorin was four times greater than that of albiflorin, as shown in Fig. 6. However, the intensities of the product ions of albiflorin are stronger than that for paeoniflorin (Fig. 6). Since paeoniflorin and albiflorin only differ in terms of an acetal or a carboxylic ester group, the higher ionization efficiency of albiflorin can be attributed to the carboxylic ester group.

The concentration of paeoniflorin was four times higher than albiflorin. *The peak at *m*/*z* 271 was generated from the fragmented aglycone and glucose moiety.^10)^

For both analytes, the product ions at *m*/*z* 341 and 381 were produced by the loss of glucosyl and benzoyl groups, respectively. In the MALDI-MS/MS spectrum of albiflorin, the product ion at *m*/*z* 341 was the most dominant. This is due to the stability of the sodium adduct that was formed, which was facilitated by the carboxylic ester formed after the cleavage of the glycosyl bond. Meanwhile, the paeoniflorin spectrum contained a product ion at *m*/*z* 219 with a relative intensity that was greater than those of the product ions at *m*/*z* 341 and 381. This is because the product ions were labile due to the absence of the carboxylic ester group in paeoniflorin. The aforementioned results confirm that the carboxylic ester group has a higher affinity for a sodium cation than the ester group during ionization.

## CONCLUSIONS

The neighboring hydroxyl groups, which are generally polarized, were found to mediate the ester fragmentation in 4′-galloyl paeoniflorin and 4-galloyl albiflorin. A proton is required during the ester fragmentation, and one is readily available from the neighboring hydroxyl group of the glucose moiety. Thus, the neighboring hydroxyl groups play a key role in the fragmentation of esters.

Comparing the ionization efficiencies of the functional isomers of albiflorin and paeoniflorin, the carboxylic ester group had a higher affinity for sodium compared with the acetal group in sodium adducts. The carbonyl oxygen is negatively polarized, thus having Lewis basicity characteristics. The sodium adduct at the carboxylic ester is more stable than that for the acetal derivative.

## Notes

Mass Spectrom (Tokyo) 2022; 11(1): A0101
